# Genetic Diversity and Population Structure Analysis of Soybean [*Glycine max* (L.) Merrill] Genotypes Using Agro-Morphological Traits and SNP Markers

**DOI:** 10.3390/genes15111373

**Published:** 2024-10-25

**Authors:** Felicity Kido Chiemeke, Bunmi Olasanmi, Paterne A. Agre, Hapson Mushoriwa, Godfree Chigeza, Abush Tesfaye Abebe

**Affiliations:** 1 Pan African University Life and Earth Science Institute (Including Health and Agriculture), Ibadan 200132, Oyo, Nigeria; chiemekefelicity@gmail.com; 2 Department of Crop and Horticultural Sciences, Faculty of Agriculture, University of Ibadan, Ibadan 2000113, Oyo, Nigeria; bunminadeco@yahoo.com; 3 International Institute of Tropical Agriculture, Oyo Road, P.M.B. 5320, Ibadan 200001, Oyo, Nigeria; p.agre@cgiar.org (P.A.A.); h.mushoriwa@cgiar.org (H.M.); 4 International Institute of Tropical Agriculture, Southern Africa Research and Administration Hub (SARAH) Campus, Lusaka 10101, Zambia; g.chigeza@cgiar.org

**Keywords:** soybean, SNP Marker, grain yield, genetic diversity

## Abstract

**Background/Objectives:** Understanding the genetic diversity of soybean genotypes can provide valuable information that guides parental selection and the design of an effective hybridization strategy in a soybean breeding program. In order to identify genetically diverse, complementary, and prospective parental lines for breeding, this study set out to ascertain the genetic diversity, relationships, and population structure among 35 soybean genotypes based on agro-morphological traits and Single Nucleotide Polymorphic (SNP) marker data. **Methods/Results**: Cluster analysis, based on agro-morphological traits, grouped the studied genotypes into four clusters. The first two principal components accounted for 62.8% of the total phenotypic variation, where days to 50% flowering, days to 95% maturity, grain yield, shattering score, and lodging score had high and positive contributions to the total variation. Using the SNP marker information, mean values of 0.16, 0.19, 0.067, and 0.227 were obtained for minor allele frequency (MAF), polymorphic information content (PIC), observed heterozygosity (Ho), and expected heterozygosity (He), respectively. Using different clustering approaches (admixture population structure, principal component scatter plot, and hierarchical clustering), the studied genotypes were grouped into four major clusters. **Conclusions**:The agro-morphological and molecular analysis results indicated the existence of moderate genetic diversity among the studied genotypes. The traits identified to be significantly related to yield provide valuable information for the genetic improvement of soybeans for yield.

## 1. Introduction

Soybean is regarded as a “miracle crop” because it is a primary source of both oil and protein [1,2,3,4]; it is the fourth most extensively cultivated crop in the world [1,3]. In terms of overall production and commerce, soybeans rank among the world’s principal oil crops and legumes [5,6]. Soybean [*Glycine max* (L.) Merrill] has 20 chromosomal pairs with a genome of about 1100 megabases (Mb) in size [7]. It is a self-pollinated crop and hence has low allelic diversity [3,4]. In comparison to other main oilseeds, like rapeseed, sunflower, and peanut, which are expected to grow at 1.4% annually, soybean production is predicted to grow at a faster pace of 1.6% yearly over the next 10 years [1]. Africa accounts for about 2% of the world’s soybean production, with South Africa, Nigeria, and Zambia as its major producers [1,8]. This crop has great potential to improve the nutritional health of low-income communities in Sub-Saharan Africa (SSA), offering an excellent source of protein and essential nutrients [1,9,10]. Soybean roots, as a leguminous crop, also fix nitrogen in the soil through symbiosis with Rhizobium bacteria. This contributes to improved soil fertility, resulting in more sustainable cereal production in rotations, making it a profitable option for SSA agricultural systems, particularly for smallholder farmers [1,11,12]. Thus, it is a crop with a strong potential for expansion in SSA, and its ongoing demand is principally driven by the expanding feed sector for poultry, aquaculture, and domestic consumption [2]. Approximately 170,000 soybean germplasm have been conserved worldwide across 17 nations in order to preserve its genetic diversity, while the Chinese National Crop Genebank has 31,575 accessions [5]. These multiple distinct germplasm collections served as sources of genes that can be used to improve the crop’s genetic makeup and have significantly aided production and breeding efforts [5].

Based on available evidence, soybean cultivation as a commercial crop in Africa dates back as early as 1903 in South Africa, 1907 in Tanzania, 1908 in Nigeria, and 1909 in Malawi [1,2,13]. Among grain legumes, soybean is in great demand due to its importance in animal feed, human diet, and industrial products such as biodiesel manufacture [14,15]. It is primarily produced in Nigeria for its seeds and used to make soymilk, bean curd, soy soups, soy ogi, and tom brown, among other delicacies [16]. It serves as a cheap source of plant protein in the diets of many Nigerians, particularly children [16]. Due to its ever-increasing demand as a key cash crop for rural households in Nigeria and other Sub-Saharan Africa (SSA) regions, concerted efforts are greatly required to improve yields [17,18].

Studies have established that soybean varieties with considerable yield performance are needed to sustain production, but their improvement has been limited by low genetic diversity [3,19,20]. Its highly limited genetic base has posed a challenge to the genetic improvement of such an important crop [1,5,21]. Breeding programs always strive to increase the genetic diversity of crops, thereby boosting the potential of populations to develop new varieties [3,22]. Understanding soybean genetic diversity and structure can aid in designing breeding strategies that ensure the development and identification of high-yielding and well-adapted soybean varieties that enhance the yield and production of the crop [23]. One of the earliest and most widely used techniques in genetic diversity analyses is based on phenotypic traits, regarded as the best determinant of taxonomic categorization and agronomic usefulness of agricultural plants [24]. Notwithstanding the success of these approaches, they are not always sufficiently informative, especially when the features are very sensitive to genotype-by-environment interactions. This has spurred academics to develop other methodologies, such as DNA-based marker analysis [1,25].

Molecular markers are very trustworthy genetic tools that can support phenotypic description in breeding programs [25]. Over the last few decades, molecular markers used to define genetic variation have evolved from Restriction Fragment Length Polymorphisms (RFLPs) to Simple Sequence Repeats (SSRs) and then to next-generation sequencing of Single Nucleotide Polymorphisms (SNPs) [26]. Nonetheless, due to the high number of markers that can be produced at a low cost, SNPs are increasingly becoming the preferred markers for genetic study and breeding. Additionally, SNPs are the most common sources of variation in eukaryotic genomes and variant calling and are more accurate due to their bi-allelic nature [23,27,28]. Increased genetic diversity may be largely attributed to populations formed by genetic recombination of biparental crossings of different parents [3,5,29]. Therefore, the objective of this study was to determine and assess the extent of genetic diversity of these genotypes using morphological traits and molecular markers.

## 2. Materials and Methods

### 2.1. Plant Materials

In this study, a total of thirty-five genotypes (Table 1) sourced from the soybean breeding program of the International Institute of Tropical Agriculture (IITA), Ibadan, Oyo State, Nigeria, were evaluated.

### 2.2. Experimental Sites and Design

The field experiment was conducted on three trial sites of the International Institute of Tropical Agriculture (IITA), i.e., Ibadan, Oyo State; Zaria, Kaduna State; and Ikenne, Ogun State of Nigeria.

The IITA Ibadan trial field is located at a latitude of 7°30′ N, a longitude of 3°54′ E, and an altitude of 243 masl. The annual rainfall ranges from 1300 to 1500 mm, and the minimum daily temperature ranges between 21 °C and 23 °C, while the maximum temperature is between 28 °C and 34 °C. The test locations represent the humid tropical lowland soybean-growing agroecology of Nigeria.

The site at Zaria is located at a longitude of 7°42′ E, a latitude of 11°04′ N, and an altitude of 640 masl, with an average minimum and maximum temperature of 15 °C and 32 °C, respectively. Zaria belongs to the Northern Guinea savanna agroecological zone of Nigeria, and the annual average rainfall ranges from 500 to 1045 mm.

The Ikenne site is located at a longitude of 3°43′ E, a latitude of 6°52′ N, with 21 °C and 31 °C as the average minimum and maximum temperatures, and an altitude of 235.2 m above sea level. It has an average rainfall of 1200 mm and belongs to the humid forest of Nigeria. 

The genotypes were evaluated in a 5 × 7 alpha lattice design, each planted in a plot of 4 rows, each 4 m long. All the trials were carried out in the year 2022.

### 2.3. Data Collections

The data were collected on traits presented in Table 2.

### 2.4. Genotyping Using SNP Markers

Seeds of the 35 genotypes were sown in the screen house at IITA, Ibadan, for sampling. However, seeds of two genotypes did not germinate, probably due to poor viability and other environmental factors. For the analysis, immature, healthy trifoliate leaves from three-week-old plants were collected from four to five plants of each of the remaining 33 genotypes (Table 1) and kept in a zip-lock bag on ice and later stored at −80 °C in a deep freezer dryer. Prior to genomic DNA extraction, each sample leaf was bulked and lyophilized for 72 h in a Labconco Freezone 2.5 L System lyophilizer (Marshall Scientific, LABCONCO, Kansas, MO, USA) and reduced to a fine powder in the SpexTM Sample Prep 2010 Geno/Grinder (Thomas Scientific, Metuchen, NJ, USA).

### 2.5. DNA Extraction and Genotyping Using SNP Markers

Intertek-AgriTech (http://www.intertek.com/agriculture/agritech/) accessed 16 January 2024 and LGC oKtopure^TM^ automated high-throughput ‘sbeadex^TM^’ were used for DNA extraction and purification system (https://www.biosearchtech.com/, accessed 16 January 2024), in which the magnetic separation method was used in the ‘sbeadex^TM^’ technique to prepare nucleic acids.

The first stage in this process was to homogenize leaf tissue samples in 96 deep-well plates using steel bead grinding. The ground tissue was treated with DNA extraction buffer using LGC’s ‘sbeadex^TM^’ kit for plant DNA preparation (https://www.biosearchtech.com/, accessed 16 January 2024). Later on, super-paramagnetic particles coated with ‘sbeadex^TM^’ surface chemistry that attracts nucleic acids from the plant sample were used to purify extracted DNA. Purified DNA was then extracted and used for downstream procedures.

### 2.6. Multivariate Statistical Analysis Using Agro-Morphological Traits

All the multivariate diversity techniques, i.e., cluster and principal component analyses, were performed based on the combined mean of the genotypes across the three locations for the various yield and yield-related agro-morphological traits.

#### Cluster Analysis Based on Agro-Morphological Traits

Hierarchical clustering methods are commonly employed in the analysis of genetic diversity in crop species. The best linear unbiased estimates (BLUES) of the agro-morphological data were used to analyze the cluster dendrogram. The dendextend version is 1.17.1. R software version 4.3.1 of R Core Team 2023 was used for the analyses.

Principal Component Analysis (PCA) was performed to determine the contribution of the traits to the observed variations among the genotypes. In this PCA, only PCs (Principal Components) with eigenvalues greater than one were retained. The analysis was carried out using Factoextra version 1.0.7. in R software version 4.3.1 of R Core Team 2023 [30].

### 2.7. Analysis of SNP Markers Summary Statistics

The generated raw files from the DArT were transformed to hapmap and later to variant call format (VCF). Low-quality markers such as minor allele frequencies <0.01, poorly read depth <5, genotype quality <20, unmapped markers to any chromosome, and duplicated markers were eliminated using the software PLINK 1.9 [31] and vcftools [32]. Finally, a total of 10,630 relevant SNP markers distributed across the 20 soybean chromosomes were retained and used for further analysis (Table S2). Using Plink and vcftools, genetic parameters of SNPs, such as MAF, PIC, and Ho and He, were estimated.

#### Population Structure

The population structure analysis was performed using ADMIXTURE version 1.3.0 [33]. The k-means analysis was used to determine the optimal number of clusters by varying the number of clusters from 1 to 10. The appropriate K value was determined using cross-validation error [34]. In the admixture analysis, genotypes with membership proportions (Q-value) ≥60% were assigned to groups. In comparison, genotypes with ancestry probability <60% were considered admixed [35]. A complementary approach based on PCA and hierarchical cluster analyses was used to understand the relationship among the 33 genotypes. Principal component analysis (PCA) was then conducted to determine the genetic relationships among the evaluated soybean genotypes using FactoMineR [36] and FactoExtra R packages [37]. The Jaccard dissimilarity matrix using the phylentropy R package was used to establish the hierarchical cluster (HC) [38], and a dendrogram was plotted using the Ward D2 method.

## 3. Results

### 3.1. Cluster Analysis

**Figure 1:**
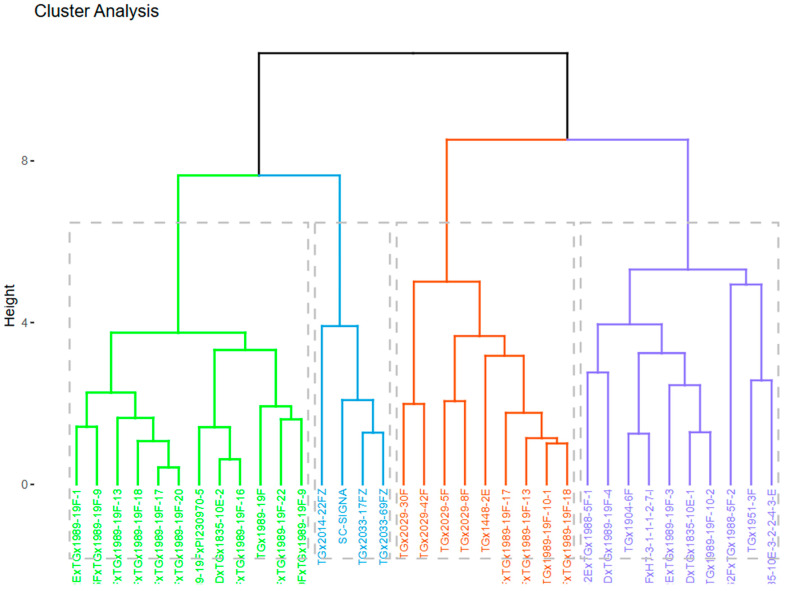
Cluster dendrogram of thirty-five soybean genotypes evaluated across three locations in Nigeria in 2022 based on agro-morphological traits.

The cluster dendrogram, based on seven agro-morphological traits of the 35 soybean genotypes, resulted in four major cluster groups (Figure 1). The genotypes grouped in each cluster based on the quantitative traits are shown in Table 3, while the mean and standard deviation of the various traits in each cluster group are presented in Table 4. Cluster I comprised four genotypes depicting a cluster mean plant height of 77.9 cm, highest hundred seed weight (17.3 g), earliest days to 50% flowering (46 days), 113 days to 95% maturity, second highest grain yield of 2685 kg/ha and second lowest lodging (1.69) and shattering (1.71) scores. Cluster II contained nine genotypes with the highest cluster mean value for days to 95% maturity (120 days), days to 50% flowering (51 days), and the lowest cluster mean for lodging score (1.28), shattering score (1.54), and grain yield (2319 kg/ha). Cluster III comprised ten genotypes with the latest days to 95% maturity (120 days), days to 50% flowering (51), the highest lodging score (2.65), and the second highest shattering score (2.79) cluster mean value. The cluster had the lowest 100 seed weight of 12.2 g and a grain yield of 2350 kg/ha. Cluster IV contained twelve genotypes, with a cluster mean grain yield of 2773 kg/ha and the highest shattering score of 2.91. The cluster also displayed the second lowest 100 seed weight (14 g) and days to 95% maturity (117 days); shortest plant height (65.5 m); and lodging score of 2.00. Since cluster analysis grouped genotypes with higher morphological similarity together, representative accessions from a certain cluster may be selected for hybridization with genotypes from another cluster.

### 3.2. Principal Component Analysis (PCA)

The PCA revealed two principal components (PCs) with eigenvalues >1 that accounted for 62.8% of the total variation among the studied genotypes (Table 5). PC1 contributed to 34.6%, while PC2 contributed to 28.2% of the total variation. The traits that had a high positive contribution to PC1 were days to 50% flowering (0.498) and days to 95% maturity (0.522). Plant height, grain yield, and 100 seed weight showed a high negative contribution to PC1. PC2 was highly and positively correlated with grain yield (0.417), lodging score (0.538), and shattering score (0.637), while days to 95% maturity (−0.150), plant height (−0.290), and hundred seed weight (−0.010) were negatively associated.

### 3.3. Principal Component Biplot

**Figure 2:**
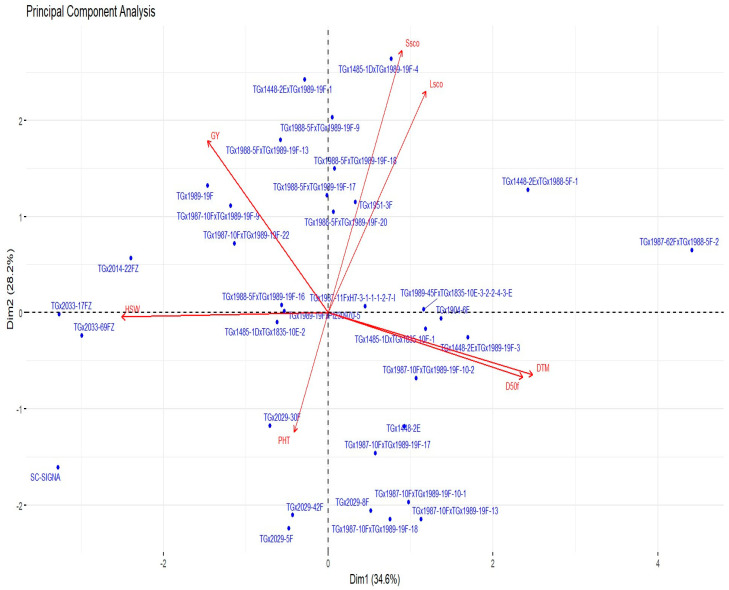
Biplot of the first two principal components showing the 35 genotypes and their association with the seven phenotypic traits. In this figure, GY: grain yield in kg/ha, HSW: hundred seed weight in g, PHT: plant height, D50f: days to fifty percent flowering, DTM: 95% days to maturity, Lsco: lodging score, Ssco: shattering score.

Days to 95% maturity and days to 50% flowering were found to be significantly and positively correlated, as well as lodging score and shattering score (Figure 2). Grain yield was positively correlated with 100 seed weight, shattering score, and lodging score. Plant height positively correlated with hundred seed weight, days to 95% maturity, and days to 50% flowering. Hundred seed weight negatively correlated with days to 50% flowering, lodging score, days to 95% maturity, and shattering score. The principal component biplot was not only able to identify traits contributing to grain yield but also indicated the outperforming genotypes as it helped select genotypes with the trait of interest. Genotypes identified to perform averagely across the seven phenotypic traits, as shown in the biplot, are located around the origin, which includes TGx 1988-5F x TGx 1989-19F-16, TGx 1989-19F, TGx1987-11FxH7-3-1-1-1-2-7-1, and TGx 1989-19F x PI230970-5. The PCA biplot showed that genotypes TGx 1988-5F x TGx 1989-19F-9, TGx 1987-10F x TGx 1989-19F-18, and TGx 1987-10F x TGx 1989-19F-9 were associated with grain yield, shattering score, and lodging score, whereas genotypes TGx 2033-17FZ and TGx 2033-69FZ were associated with 100 seed weight. The genotypes TGx 1448-2E x TGx 1989-19F-1, TGx 1989-19F, and TGx 1988-5F x TGx 1989-19F-13 were associated with high grain yield, while TGx 2029-30F and TGx 2029-42F were associated with high plant height. The genotypes TGx 1448-2E x TGx 1989-19F-3, TGx 1485-1D x TGx 1835-10E-1, and TGx 1485-1D x TGx 1835-10E-2 were associated with days to 95% maturity and days to 50% flowering and shattering score. The genotypes highly associated with lodging score and days to 95% maturity were TGx 1485-1D x TGx 1989-19F-4, TGx 1987-62F x TGx 1988-5F-2, and TGx 1987-62F x TGx 1988-5F-1, while SC-Signa was associated with 100 seeds weight and plant height.

### 3.4. Markers Summary and Population Structure

A filtered total of 10,630 SNPs distributed across twenty (20) chromosomes of the soybean genome were retained for molecular analysis. The distribution summary of the genetic parameters is presented in Table 6. The minor allele frequency had an average value of 0.162, ranging from 0.125 in chromosome7 to 0.200 in chromosome14. The mean value for the observed heterozygosity was 0.06, ranging from 0.049 in chromosome10 to 0.079 in chromosome13, while the average value of expected heterozygosity was 0.227, ranging from 0.179 in chromosome10 to 0.269 in chromosome14. The polymorphic information content (PIC) had a mean value of 0.185, varying from 0.153 in chromosome7 to 0.210 in chromosome1.

**Figure 3:**
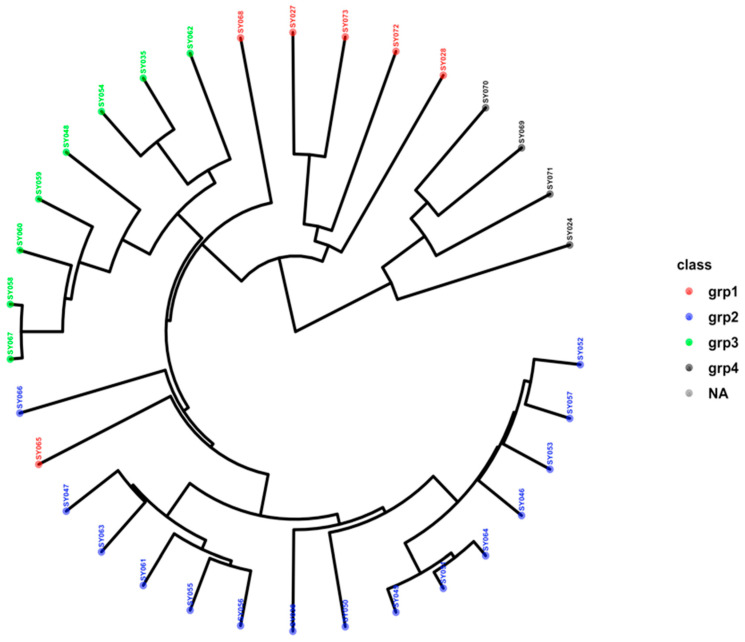
Hierarchical clustering dendrogram of thirty-three soybean genotypes based on 10,630 SNP markers generated using the Ward D2 method and Jaccard’s dissimilarity matrix.

The hierarchical clustering based on SNP markers grouped the 33 genotypes into four major groups, which were differentiated by their respective colors, with group I containing six genotypes, group II having fifteen genotypes, group III containing eight genotypes, and group IV with four genotypes. Genotype SY065 belonging to group I was clustered with group II and, therefore, has 16 member genotype clusters; group I has a five-member genotype cluster, group III has an eight-member genotype cluster, and group IV has a four-member genotype cluster (Figure 3).

**Figure 4:**
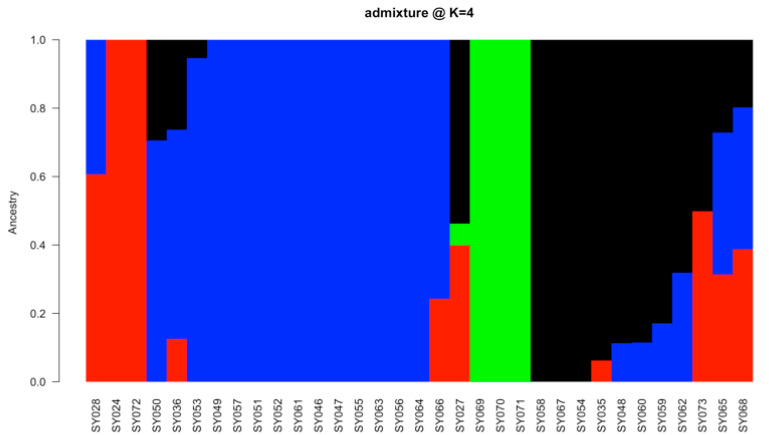
Population structure based on admixture analysis of thirty-three soybean genotypes. Subpopulations were set at k = 4. The colors represent the four clusters: Cluster 1 (red), cluster 2 (blue), cluster 3 (green), and cluster 4 (black) based on a membership coefficient of ≥60%, admixed membership based on a coefficient of <60%.

Admixture population structure analysis, at a minimum value of *k* equal to four based on cross-validation error (CV error), discriminated the soybean genotypes into four clusters (Figure 4). Genotypes with membership coefficients >0.60 were assigned to the corresponding pure groups, which made up the four groups represented by different colors, with group 1 (green) having three genotypes, group 2 (blue) with fifteen genotypes, group 3 (red) with four genotypes, and group 4 (black) having eight genotypes. Those with coefficients <0.60 were assigned to be admixt in the population structure. Admixed genotypes included SY073, SY065, and SY068 (G1, G4, and G17).

**Figure 5:**
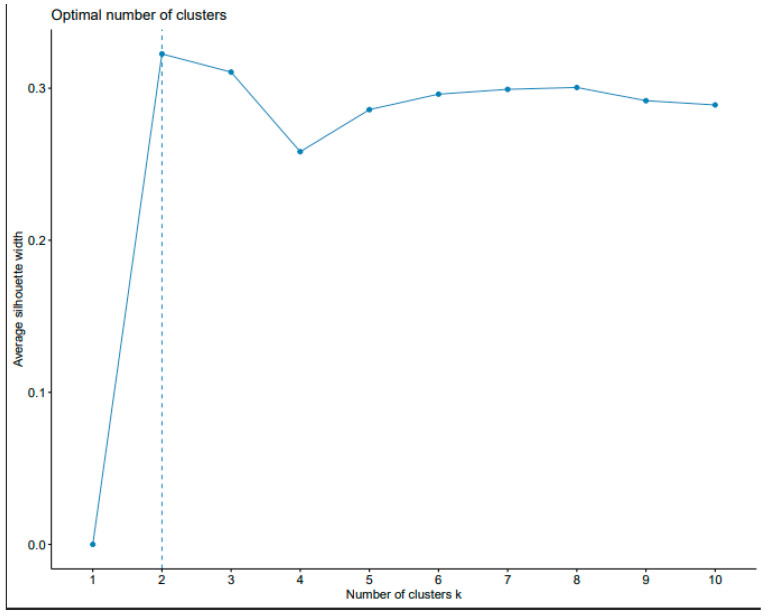
Silhouette width optimum clustering.

**Figure 6:**
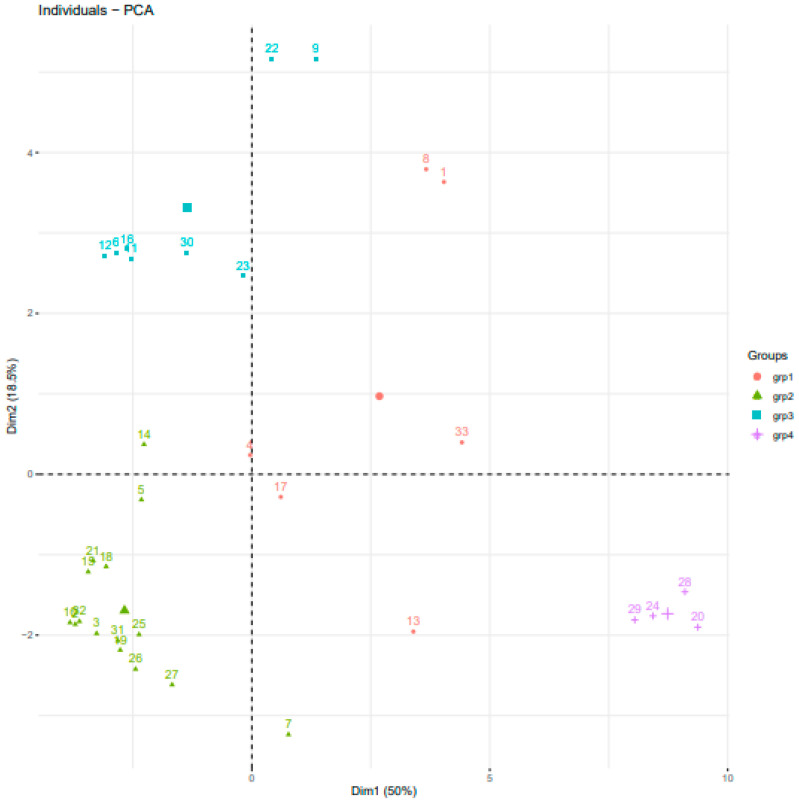
Scatter plot of individuals on the first two principal components analysis of thirty-three soybean genotypes using 10,630 SNP markers.

Through silhouette K-means analysis, the optimum number of clusters was identified to be 2 (Figure 5), with a sub-population obtained at K = 4. (Figure 6 of the PCA); the first two PCs explained a total cumulative molecular variation of 68.5%, with PC1 and PC2 accounting for 50% and 18.5% of the total genetic variation, respectively, were subgrouped into four clusters of five, sixteen, eight, and four genotypes in each using the discriminant analysis (Figure 6) as genetically related individuals. The principal components (PCs) established the stability of the potential population structure. The above results were consistent and exhibited good uniformity, as summarized in Table 7, showing that the sample population structure was appropriately identified.

## 4. Discussion

Cluster analysis grouped the study genotypes together with high similarity within a cluster group and dissimilarity among cluster groups. Genotypes within the cluster showed less variation, whereas genotypes in different clusters showed more diversity. As a result, genotypes from different and distant clusters could be used as parental lines in crossing programs to improve the traits. This helps to choose the most diverse parental lines possible to ensure high genetic recombination and transgressive segregation in the progeny population. Darai et al. [39] studied the genetic diversity of 104 soybean genotypes and reported five cluster groups; Dubey et al. [40], who assessed 50 soybean genotypes, reported 10 clusters; Iqbal et al. [41], who studied 135 soybean genotypes and reported five clusters; Mofokeng [42]; Singh and Shrestha [43] evaluated 20 soybean genotypes and reported five clusters; Sivabharathi et al. [4] assessed 135 soybean genotypes and reported 12 clusters; Vijayakumar et al. [44] evaluated 50 soybean genotypes and reported eight clusters, while Zafar et al. [45] evaluated 123 soybean genotypes and reported 17 clusters.

The first two PCs [based on metric (quantitative) traits] with Eigen values greater than one and a cumulative contribution of 62.8% of the total variation were selected as the important PCs. Vijayakumar et al. [44] reported 73.7% of the total variation by the first three major PCs, while Singh and Shrestha [43] reported 84.1% contribution by the first four PCs. Sivabharathi et al. (2023) reported a 79.8% major contribution by the first four PCs, and Kujane et al. [24] recorded a 45.4% major contribution by the first three PCs. In the current study, the first PC that contributed to 34.6% was positively associated with days to 95% maturity and days to 50% flowering. This result concurs with the findings of Singh and Shrestha [43] and Denwar et al. [46], who reported days to 95% maturity and days to 50% flowering to be positively associated with PC1. The second PC that contributed to 28.2% of the total variation was mainly associated with grain yield, lodging score, and shattering score. Darai et al. [39] and Jain et al. [47] reported a similar association of PC2 with grain yield. The strong positive and significant correlation found between days to 50% flowering and days to 95% maturity in the PCA biplot was similarly reported by Denwar et al. [41]. The PCA biplot can be easily utilized in the identification of genotypes that are outperforming based on selection interest. In this study, TGx 1989-19F and TGx1988-5FxTGx1989-19F-13 were both associated with grain yield. Similarly, utilized as in the report given by Kujane et al. [24] and Denwar et al. [46], who reported genotypes PR-165-52, B 66 S 8, Dundee, and N 69-2774 were associated with high oil content, and LD 15-2224, LD 11-2170, LG 14-6201, and LD 14-3214 were associated with 100 seed weight, respectively.

Additionally, molecular profiling has emerged as a preferred option in genetic diversity studies due to its reliability and authenticity. Lower heterozygosity and allelic diversity are commonly expected in a population of self-fertilizing species such as soybeans [1,5,48]. The PIC values range from 0.153 to 0.210 with an approximate mean value of 0.2, which can be regarded as moderately informative and implies that the SNP markers have differentiating power since PIC cannot exceed 0.50 in bi-allelic markers [1]. The mean PIC of 0.185 recorded in this study is nearly similar to the 0.199 PIC reported by Bisen et al. [49] based on 16 SSR markers in 38 soybean genotypes and lower than 0.29 reported by Lukanda et al. [3] using 6395 SNPs in 282 soybean accessions. The Ho (0.067) in this study is similar to the 0.066 Ho reported by Liu et al. [50] based on 5195 SNPs in 277 Chinese soybean accessions. Observed heterozygosity found in this study ranged from 0.049–0.079, with an average of 0.067, implying the existence of high genetic variability among the studied genotypes. It is higher than the 0.058 reported by Chander et al. [1] based on the 186 SNPs in 155 soybean accessions and 0.050 reported by Lukanda et al. [3] based on the 6395 SNPs in 282 soybean accessions. In other crops, such as Barley, Yirgu et al. [28] reported a Ho of 0.045 based on a study made using 10,103 SNPs in 105 Ethiopian Barley genotypes. In contrast, the Ho reported in this study was lower than the 0.193 reported by Abebe et al. [5] in the diversity study of 65 soybean genotypes based on SNP markers and 0.33 reported by Lukanda et al. [3] using 6395 SNPs in 282 soybean accessions. Minor allele frequency (MAF) ranged from 0.125 to 0.200 with a mean value of 0.162, implying that further valuable genes can be exploited from the genotypes used in this study [28]. It was lower than the 0.268 MAF reported by Liu et al. [50] using 5195 SNPs, 0.23 reported by Abebe et al. [5] using SNPs in 65 soybean genotypes, and 0.22 reported by Lukanda et al. [3].

The average genetic similarity among a set of genotypes measures genetic diversity at the population level [5]. The admixture population structure, DAPC-based principal component scatter plots, and the hierarchical complementary clustering analysis employed in this study differentiated the 33 soybean genotypes from each other, assigning them into four different groups, which shows substantial genetic diversity. Abebe et al. [5], using each of the structural population analyses of admixture ancestry, DAPC clustering, and hierarchical clustering methods, revealed a consistent grouping of the three clusters, each using SNPs, in the study of 65 soybean genotypes. Also, Liu et al. [50] reported two clusters based on a principal component scatter plot, neighbor-joining tree, and population structure with an optimum cluster number (K) of two in the study of 577 soybean accessions using SNP markers.

As presented in Table 6, there is a high level of correspondence between the type and number of genotypes clustered by DAPC and HC, while the level of correspondence between the admixture population structure with each of DAPC and HC clustering was moderate. This might indicate that the DAPC and HC clustering can be used interchangeably for the interpretation of the clustering in this study, as they provided highly similar clustering. There was a very low level of correspondence of the type and number of genotypes between the agro-morphological traits and genetic-based clustering. This low level of correspondence might be due mainly to the effects of environment and GXE interaction effect of the studied genotype traits across locations.

As a result, these findings suggest that there is genetic variation among the genotypes and show that the markers chosen were insightful and helpful for future research on soybean genetic diversity.

## 5. Conclusions

In the current study, the genetic diversity among some soybean genotypes was evaluated using both the multivariate analysis of phenotypic traits and single nucleotide polymorphism markers to select genetically complementary and promising parental lines for breeding programs. Variation was observed among genotypes across the studied traits and the SNP genetic data. Population structure, complimented with the discriminant analysis principal component scatter plot and hierarchical cluster analysis, as well as the cluster dendrogram multivariate analysis, has all identified four major groups. This indicates that the soybean genotypes were diverse and, hence, can be utilized for selection in future plant breeding programs.

Based on the mean values among the genotypes for the studied traits, most especially grain yield, Custer I and IV could be used to select diverse and complementary parental lines for hybridization.

## Figures and Tables

**Table 1:** The pedigree and code of thirty-five soybean genotypes evaluated at three locations in Nigeria in 2022.

S/No.	Pedigree	Genotype Code	SNP Designation
1	TGX1987-10F ×TGX1989-19F-10-1	G1	SY073
2	TGX1988-5F × TGX1989-19F-17	G2	SY049
3	TGX1485-1D × TGX1989-19F-4	G3	SY057
4	TGX1987-10F × TGX1989-19F-10-2	G4	SY065
5	TGX1448-2E × TGX1989-19F-3	G5	SY050
6	TGX1987-10F × TGX1989-19F-17	G6	SY058
7	TGX1987-10F × TGX1989-19F-22	G7	SY066
8	TGX1987-10F × TGX1989-19F-9	G8	SY027
9	TGx1987-11F×H7-3-1-1-1-2-7-I	G9	SY035
10	TGX1988-5F × TGX1989-19F-16	G10	SY051
11	TGX1988-5F × TGX1989-19F-18	G11	SY059
12	TGX1987-10F × TGX1989-19F-13	G12	SY067
13	TGX1485-1D × TGX1835-10E-2	G13	SY028
14	TGX1988-5F × TGX1989-19F-20	G14	SY036
15	TGX1448-2E × TGX1989-19F-1	G15	SY052
16	TGX1988-5F × TGX1989-19F-13	G16	SY060
17	TGX1448-2E × TGX1988-5F-1	G17	SY068
18	TGX1988-5F × TGX1989-19F-9	G18	SY053
19	TGX1987-10F × TGX1989-19F-18	G19	SY061
20	TGx1989-45F×TGx1835-10E-3-2-2-4-3-E	G20	SY069
21	TGX1987-62F × TGX1988-5F-2	G21	SY046
22	TGX1485-1D × TGX1835-10E-1	G22	SY054
23	TGX1989-19F x PI230970-5	G23	SY062
24	TGx 2029-8F	G24	SY070
25	TGx 2029-30F	G25	SY047
26	TGx 2029-42F	G26	SY055
27	TGx2029-5F	G27	SY063
28	TGx 2014-22FZ	G28	SY071
29	TGx 2033-17FZ	G29	SY024
30	TGx 2033-69FZ	G30	SY048
31	TGx 1951-3F	G31	SY056
32	TGx 1448-2E	G32	SY064
33	SC-SIGNA	G33	SY072
34	TGx 1989-19F	G34	N/A
35	TGx 1904-6F	G35	N/A

**Table 2:** Data collection method and trait description.

Traits	Code	Collection Methods	Period of Collection (Vegetative or Harvesting)	Nature of the Traits (Qualitative or Quantitative)
Plant height (cm)	PHT	It is measured from the soil surface to the tip of the plant at maturity using a meter ruler.	Harvesting	Quantitative
Days to 50% flowering	D50F	Counted from the date of sowing to the date when 50% of the plants in a plot have at least one open flower.	Vegetative	Quantitative
Days to 95% maturity	DTM	Recorded as the number of days from sowing to the date when 95% of the pods in a plot turned to their mature color.	Harvesting	Quantitative
Stem lodging score	LSCO	Collected as the average lodging of plants in each plot on a 1 to 5 scale with 1 = plants fully erect and 5 = all plants prostrate observed from the main plot.	Harvesting	Quantitative
Shattering score	SSCO	Scoring was conducted two weeks after maturity when the pods were rated for shattering on a 1 to 5 scale, with 1 = no shattering and 5 = 100% of the pods shattered.	Harvesting	Quantitative
100 seed weight (g)	HSW	It was measured by weighing one hundred seeds from plants selected at random and using a digital weighing balance, the weight in grams (g) was recorded.	Harvesting	Quantitative
Grain yield (Kg/ha)	GY	It was measured as the net plot grain weight that is used to estimate yield/hectare.	Harvesting	Quantitative

**Table 3:** Soybean genotypes in each cluster are based on agro-morphological traits.

Cluster Name	Number of Genotypes	Genotype Codes
Cluster I	4	G28, G33, G29 and G30
Cluster II	9	G25, G26, G27, G24, G32, G6, G12, G1 and G19
Cluster III	10	G17, G3, G35, G9, G5, G22, G4, G21, G31 and G20
Cluster IV	12	G15, G18, G16,G11, G2, G14, G23, G13, G10, G34, G7 and G8

**Table 4:** Mean values and standard deviation for seven traits among soybean genotypes in each cluster.

Traits	Cluster I	Cluster II	Cluster III	Cluster IV	*p*-Value
	N = 4	N = 9	N = 10	N = 12	
DTM	113 (1.29)	120 (2.75)	120 (3.75)	117 (1.88)	0.001
PHT	77.9 (8.88)	75.7 (12.2)	74.1 (13.3)	65.5 (6.29)	0.082
D50f	45.7 (0.31)	51.0 (2.71)	51.0 (2.68)	48.9 (1.00)	0.001
GY	2685 (304)	2319 (206)	2350 (201)	2773 (195)	<0.001
HSW	17.3 (0.86)	13.3 (0.85)	12.2 (1.32)	14.0 (0.82)	<0.001
LSCO	1.69 (0.60)	1.28 (0.20)	2.65 (0.44)	2.00 (0.37)	<0.001
SSCO	1.71 (0.58)	1.54 (0.31)	2.79 (0.55)	2.91 (0.40)	<0.001

Where N: number of genotypes in the cluster, DTM: days to 95% maturity, PHT: plant height in cm, D50f: days to 50% flowering, GY: grain yield, HSW: hundred seed weight in g, LSCO: lodging score, SSCO: shattering score, *p*-value: probability value (≤1%).

**Table 5:** Eigenvectors, eigenvalues, and contribution of traits to the observed variation in each principal component.

Trait	PC1	PC2
DTM	0.522	−0.150
PHT(cm)	−0.087	−0.290
D50F	0.498	−0.156
GY(Kg/ha)	−0.309	0.417
HSW(g)	−0.528	−0.010
LSCO	0.250	0.538
SSCO	0.188	0.637
Eigenvalue	2.422	1.973
Proportion of Variance	0.346	0.282
Cumulative Proportion	0.346	0.628
% Cumulative	34.6	62.8

Where PC: principal component, DTM: days to 95% maturity, PHT: plant height, D50f: days to fifty percent flowering, GY: grain yield, HSW: hundred seed weight, LSCO: lodging score, SSCO: shattering score.

**Table 6:** Summary statistics of genetic diversity parameters across 20 chromosomes of soybean using 10,630 SNP markers.

Chromosomes	SNPs Number	MAF	PIC	Ho	He
1	356	0.191	0.210	0.078	0.259
2	499	0.146	0.172	0.070	0.209
3	508	0.172	0.196	0.065	0.240
4	448	0.162	0.194	0.065	0.235
5	382	0.147	0.182	0.056	0.218
6	669	0.147	0.175	0.056	0.212
7	485	0.125	0.153	0.053	0.184
8	642	0.143	0.172	0.066	0.208
9	589	0.165	0.184	0.058	0.227
10	439	0.127	0.148	0.049	0.179
11	381	0.182	0.196	0.061	0.243
12	348	0.182	0.196	0.068	0.242
13	690	0.176	0.197	0.079	0.243
14	556	0.200	0.217	0.074	0.269
15	649	0.172	0.196	0.069	0.239
16	719	0.173	0.197	0.072	0.241
17	449	0.163	0.187	0.078	0.228
18	846	0.172	0.190	0.072	0.235
19	467	0.154	0.180	0.077	0.219
20	508	0.136	0.158	0.058	0.192
Total/Average	10630	0.162	0.185	0.067	0.227

MAF = minor allele frequency, PIC = polymorphic information content, Ho = observed heterozygosity, He = expected heterozygosity.

**Table 7:** Summary of agro-morphological traits and SNPS genetic parameter clustering/grouping of the genotypes.

Methods	I	II	III	IV
Clustering based on agro-morphological traits	G28, G29, G30, G33	G1, G6, G12, G19, G24, G25, G26, G27, G32	G3, G4, G5, G9, G17, G20, G21, G22, G31, G35	G2, G7, G8, G10, G11, G13, G14, G15, G16, G18, G23, G34
Admixture population structure	G13, G29, G33	G2, G3, G5, G7, G10, G14, G15, G18, G19, G21, G25, G26, G27, G31, G32	G8, G20, G24, G28	G6, G9, G11, G12, G22, G23, G30
DAPC	G8, G1, G33, G17, G13	G5, G14, G15, G18, G21, G3, G32, G10, G2, G31, G25, G19, G26, G27, G7, G4	G6, G11, G12, G16, G30, G23, G22, G9	G29, G24, G28, G20
HC	G1, G8, G13, G17, G33	G2, G3, G5, G7, G10, G14, G15, G18, G19, G21, G25, G26, G27, G31, G32, G4	G6, G9, G11, G12, G16, G22, G23, G30	G20, G24, G28, G29

DAPC: Discriminant analysis principal components, HC; hierarchical clustering.

## Data Availability

The original contributions presented in the study are included in the article/Supplementary Material, further inquiries can be directed to the corresponding author.

## Supplementary Materials

The following supporting information can be downloaded at: https://www.mdpi.com/article/10.3390/genes15111373/s1, Table S1: Q-Value at ≥60%, and <60% Allocation, Table S2: Mapped information across the relevant 10,630 filtered SNP markers, Table S3: Jaccard dissimilarity matrices.
