# Supplementary material for: Genetic Diversity and Population Structure Analysis of Soybean [Glycine max (L.) Merrill] Genotypes Using Agro-Morphological Traits and SNP Markers
Source: Genes (Basel). 2024 Oct 25;15(11):1373. doi: 10.3390/genes15111373 (PMC11593782; doi:10.3390/genes15111373)
Supplement: Supplementary file 1 [file genes-15-01373-s001.zip › Table S1.pdf]

Table S1: Q-Value at ≥60% and <60% Allocation

Lines	CV1	CV2	CV3	CV4	group
SY028	0.606472	0.393508	0.00001	0.00001	1
SY024	0.99997	0.00001	0.00001	0.00001	1
SY072	0.99997	0.00001	0.00001	0.00001	1
SY050	0.00001	0.705887	0.00001	0.294093	2
SY036	0.124897	0.612475	0.00001	0.262618	2
SY053	0.00001	0.946532	0.00001	0.053448	2
SY049	0.00001	0.99997	0.00001	0.00001	2
SY057	0.00001	0.99997	0.00001	0.00001	2
SY051	0.00001	0.99997	0.00001	0.00001	2
SY052	0.00001	0.99997	0.00001	0.00001	2
SY061	0.00001	0.99997	0.00001	0.00001	2
SY046	0.00001	0.99997	0.00001	0.00001	2
SY047	0.00001	0.99997	0.00001	0.00001	2
SY055	0.00001	0.99997	0.00001	0.00001	2
SY063	0.00001	0.99997	0.00001	0.00001	2
SY056	0.00001	0.99997	0.00001	0.00001	2
SY064	0.00001	0.99997	0.00001	0.00001	2
SY066	0.242237	0.757743	0.00001	0.00001	2
SY027	0.399367	0.00001	0.063185	0.537439	3
SY069	0.00001	0.00001	0.99997	0.00001	3
SY070	0.000016	0.00001	0.999964	0.00001	3
SY071	0.000018	0.00001	0.999962	0.00001	3

SY058	0.00001	0.00001	0.00001	0.99997	4
SY067	0.00001	0.00001	0.00001	0.99997	4
SY054	0.00001	0.00001	0.00001	0.99997	4
SY035	0.061737	0.00001	0.00001	0.938243	4
SY048	0.00001	0.112668	0.00001	0.887312	4
SY060	0.00001	0.114433	0.00001	0.885547	4
SY059	0.00001	0.169081	0.00001	0.830899	4
SY062	0.00001	0.319043	0.00001	0.680937	4
SY073	0.498428	0.00001	0.00001	0.501552	Admixt
SY065	0.313651	0.414731	0.00001	0.271608	Admixt
SY068	0.387159	0.415405	0.00001	0.197426	Admixt
